# A Four-Year Course or a Three-Year Course?

**Published:** 1904-03-15

**Authors:** Frank L. Platt

**Affiliations:** San Francisco


### A FOUR-YEAR COURSE OR A THREE-YEAR COURSE?

BY FRANK L. PLATT, D.D.S., SAN FRANCISCO.

Which shall it be, a *four*-years' course of *seven* months in each year, a *three*-years' course of *nine* months in each year, or a return to a *three*-years' course of *seven* months in each year? Around these questions centered most of the debate and interest at the recent meeting of the National Association of Dental Faculties at Buffalo, New York. The importance of the question is manifest, and it is probably well that the recent meeting could not take definite action on the subject, for it is one demanding serious consideration and not to be lightly or easily decided.

That a three-years' course of but seven months in each year had become inadequate to meet the demands of a developing profession and an extended curriculum may be taken as the principal reason for the addition of a fourth year to the course of study in our dental colleges.

What other motives of a less generous nature, such as the idea that diminished classes, consequent to the adoption of a four-years' course, would lead to the closing of some of the weaker colleges, and that the addition of another year meant an additional income of many thousands of dollars to be derived from each of the larger classes of some of the stronger colleges, need not be considered here; let us hope they had no real place in leading to the adoption of a four-years' course. Granting that three years of seven months each did not provide sufficient time in which to fit the average dental student for the practice of dentistry, has the adoption of a fourth year provided the best means of remedying the defect?

Would it not have been better to have raised the standard of admission to our colleges to a degree which would not alone have demanded a high order of intellectual training and development, but also proof of a trained artistic and mechanical sense on the part of applicants?

Would it not have been better, if after all, more time was proven to be actually needed in the college course, to have lengthened each year to a period of nine months instead of seven? To our mind, there is no room for debate as to the greater excellency of either one of these alternatives, and if the two could be combined we believe the resulting condition would so far exceed in practical value the present "four years of seven months" course of study, as to shortly convince even its most ardent supporters that it was not the remedy sought.

We do believe, however, that in a measure a course of four years of seven months each is better than a course of three years with the same number of months in each year, for it will give the student some additional training and experience. There must be taken into consideration, however, the fact that in actual time consumed in college work and associations, there is practically no difference between the present course and a course of three years of nine months each. It is admitted by all educators that a considerable portion of each recurring session is spent by students in acquiring, or *reacquiring*, habits of study and concentration of purpose, and that the longer the periods of rest or inactivity between terms, the greater is the amount of time lost in again getting actually at work. In the present course there is a vacation of five months each year, or fifteen months in four years; in a course embracing three years of nine months each there are three months' vacation each year and six months in the entire course.

In one case twenty-eight months' work and fifteen months' vacation; in the other, twenty-seven months' work and six months' vacation. It seems probable that the loss of one month's work would be but little compared with the greater loss incurred by the longer vacations.

From the fact that but fifteen percent of the colleges represented at the meeting in Buffalo reported a completed curriculum covering the four-years' course, it may be inferred that the action of adopting this course was a little premature or that it has not been taken very seriously by the Association of Dental Faculties. These questions are to be more fully discussed and perhaps settled for a time at the next regular meeting of the association. In the meantime they should be carefully considered, and if it is really felt to be imperative to have a four-years' course, it is to be hoped that none of our colleges will be so indifferent to the good of their students as to insist that seven months' work per year is just as good as nine. By no means let us return to the three years of seven months' course, but if we *must* have four years, let it be *four years of nine months each.—Pacific Gazette.*

